# Supplementary material for: Yolk-deprived Caenorhabditis elegans secure brood size at the expense of competitive fitness
Source: Life Sci Alliance. 2023 Apr 14;6(6):e202201675. doi: 10.26508/lsa.202201675 (PMC10105328; doi:10.26508/lsa.202201675)
Supplement: Supplementary file 7 [file LSA-2022-01675_TableS1.docx]

**Table S1.** Proteins decreased or increased in *ceh‑60(lst466)* and *ceh‑60(lst491)* embryos *versus* wild type, ordered by the mean fold change for *ceh‑60(lst466)* and *ceh‑60(lst491)*. Statistical significance was determined using a two‑way ANOVA with Benjamini & Hochberg multiple comparison correction (N=6 corresponding to six TMT labels). Complete list of proteins measured in wild-type, *ceh‑60(lst466)* and *ceh‑60(lst491)* embryos and underlying data are available in Data S7.

| **Decreased in *ceh-60* mutants** | | | | | | |
| --- | --- | --- | --- | --- | --- | --- |
| **UniProt ID** | **Protein** | **Gene** | **Fold change *ceh-60(lst466)*** | ***P* value** | **Fold change *ceh-60(lst491)*** | ***P* value** |
| P05690 | Vitellogenin-2 | *vit-2* | 0.000013 | < 0.0001 | 0.12 | < 0.0001 |
| Q9N4J2 | Vitellogenin-3 | *vit-3* | 0.11 | < 0.0001 | 0.10 | < 0.0001 |
| P06125 | Vitellogenin-5 | *vit-5* | 0.13 | < 0.0001 | 0.12 | < 0.0001 |
| P18947 | Vitellogenin-4 | *vit-4* | 0.21 | < 0.0001 | 0.17 | < 0.0001 |
| P18948 | Vitellogenin-6 | *vit-6* | 0.29 | 0.0002 | 0.20 | < 0.0001 |
| P55155 | Vitellogenin-1 | *vit-1* | 0.38 | < 0.0001 | 0.24 | < 0.0001 |
| Q20628 | Cytidine deaminase-2 | *cdd-2* | 0.16 | < 0.0001 | 0.65 | < 0.0001 |
| Q20615 | Downstream Of DAF-16 | *dod-23* | 0.43 | < 0.0001 | 0.43 | < 0.0001 |
| Q18990 | Pyrimidine-1 | *pyr-1* | 0.57 | 0.0273 | 0.36 | 0.0036 |
| P91398 | *C. elegans* Y-box | *cey-3* | 0.37 | < 0.0001 | 0.63 | 0.0033 |
| Q17561 | Uncharacterized protein | *aly-1* | 0.64 | 0.0235 | 0.41 | 0.0016 |
| O45819 | Glycogenin like | *gyg-2* | 0.45 | 0.0024 | 0.65 | 0.0337 |
| O61861 | Actin-related protein 2/3 complex | *arx-7* | 0.50 | 0.0005 | 0.65 | 0.0104 |

| **Increased in *ceh-60* mutants** | | | | | | |
| --- | --- | --- | --- | --- | --- | --- |
| **UniProt ID** | **Protein** | **Gene** | **Fold change *ceh-60(lst466)*** | ***p* value** | **Fold change *ceh-60(lst491)*** | ***p* value** |
| Q9U2C3 | Ras-associated binding | *rab-35* | 3.13 | 0.0007 | 2.16 | 0.0231 |
| Q20057 | Enhancer of rudimentary homolog | *erh-2* | 3.11 | 0.0007 | 1.95 | 0.0482 |
| O45226 | 60S ribosomal protein L29 | *rpl-29* | 2.76 | 0.0006 | 2.06 | 0.0086 |
| Q9U238 | Translocon-associated protein | *trap-4* | 2.70 | 0.0003 | 1.89 | 0.0129 |
| O16458 | Related to yeast Vacuolar Protein Sorting factor | *vps-60* | 2.63 | 0.0013 | 1.93 | 0.0326 |
| P90994 | Glutathione-independent glyoxalase DJR-1.1 | *djr-1.1* | 1.99 | 0.0008 | 2.06 | 0.0006 |
| Q9N4I4 | 60S ribosomal protein L10a | *rpl-1* | 2.02 | 0.0150 | 1.83 | 0.0336 |
| Q9BKS1 | Elongin-C | *elc-1* | 2.09 | 0.0003 | 1.68 | 0.0059 |
| Q17539 | Uncharacterized protein | C01B10.8 | 1.91 | 0.0038 | 1.75 | 0.0092 |
| Q19972 | *C.elegans* chromodomain protein | *cec-4* | 2.02 | 0.0026 | 1.63 | 0.0369 |
| Q9U241 | Acyl carrier protein | Y56A3A.19 | 2.08 | < 0.0001 | 1.51 | 0.0056 |
| P16356 | Amanitin resistant | *ama-1* | 1.65 | 0.0007 | 1.90 | 0.0001 |
| O44144 | Permeable eggshell | *perm-4* | 1.85 | 0.0001 | 1.63 | 0.0006 |
| Q11183 | Thioredoxin domain-containing protein 9 | *txdc-9* | 1.85 | 0.0004 | 1.61 | 0.0026 |
| P49029 | Mago nashi homolog | *mag-1* | 1.96 | 0.0007 | 1.46 | 0.0292 |
| P91857 | Ras-associated binding | *rab-5* | 1.73 | 0.0025 | 1.66 | 0.0074 |
| O17695 | Histone deacetylase 1 | *hda-1* | 1.79 | 0.0038 | 1.53 | 0.0371 |
| O44444 | RRM domain-containing protein | C02B10.4 | 1.79 | 0.0005 | 1.44 | 0.0179 |
| P52018 | Cyclophylin | *cyn-11* | 1.69 | 0.0004 | 1.53 | 0.0032 |
| Q17949 | Deoxyhypusine hydroxylase | *dohh-1* | 1.68 | 0.0023 | 1.42 | 0.0228 |
| P34525 | Signal peptidase complex subunit 3 | *spcs-3* | 1.59 | 0.0135 | 1.49 | 0.0346 |
| G5EFF7 | Ubiquilin | *ubql-1* | 1.71 | 0.0005 | 1.34 | 0.0285 |
| Q95X44 | Vacuolar H ATPase | *vha-8* | 1.63 | 0.0001 | 1.42 | 0.0023 |
| Q20062 | Mitochondrial associated ribonuclease homolog | *marb-1* | 1.57 | 0.0004 | 1.46 | 0.0014 |
| Q18577 | Uncharacterized protein | C42D4.1 | 1.53 | 0.0128 | 1.44 | 0.0346 |
| O18154 | Tubulin alpha chain | *tba-7* | 1.47 | 0.0172 | 1.47 | 0.0198 |
| P34685 | F-actin-capping protein subunit alpha | *cap-1* | 1.56 | 0.0008 | 1.31 | 0.0227 |
| Q9XVS3 | C-type lectin domain-containing protein 87 | *clec-87* | 1.53 | 0.0001 | 1.31 | 0.0036 |
| G5ECL3 | Pre-RNA processing 21 | *prp-21* | 1.51 | 0.0004 | 1.33 | 0.0069 |
| P52821 | 40S ribosomal protein S25 | *rps-25* | 1.40 | 0.0073 | 1.38 | 0.0120 |
| Q27497 | GLC7 like Phosphatase | *gsp-1* | 1.30 | 0.0132 | 1.42 | 0.0037 |
| Q17430 | Spliceosome associated RNA binding factor | *sart-3* | 1.45 | 0.0013 | 1.24 | 0.0357 |
| Q22038 | RHO family | *rho-1* | 1.41 | 0.0035 | 1.25 | 0.0435 |
| Q9XW41 | Sorting NeXin | *snx-3* | 1.29 | 0.0083 | 1.30 | 0.0084 |
| O62246 | Coatomer subunit epsilon | *cope-1* | 1.33 | 0.0038 | 1.25 | 0.0224 |
